# The matrix of linguistic exclusions impeding career construction for D/deaf learners

**DOI:** 10.4102/ajod.v11i0.935

**Published:** 2022-06-13

**Authors:** Unati Stemela-Zali, Harsha Kathard, Maximus M. Sefotho

**Affiliations:** 1 Department of Rehabilitative Sciences, Faculty of Health Sciences, University of Fort Hare, East London, South Africa; 2 Department of Disability Studies, Faculty of Health Sciences, University of Cape Town, Cape Town, South Africa; 3 Department of Educational Psychology, Faculty of Education, University of Johannesburg, Johannesburg, South Africa

**Keywords:** D/deaf, Sign Language, education, exclusion, inclusive education, careers

## Abstract

**Background:**

The purpose of this research study was to explore how D/deaf learners in the Eastern Cape province of South Africa constructed their careers and what types of support were available for them to do so. The study found that among the support required, support for their linguistic development, particularly Sign Language acquisition, was critical in home, school and community settings.

**Objectives:**

The objective of this study was to highlight the multiple linguistic exclusions faced by D/deaf learners in the Eastern Cape, which negatively impacted their career construction.

**Method:**

Savickas’s theory of career construction framed this analytical-qualitative case study. The study was conducted in two out of four schools for the D/deaf in two districts of the Eastern Cape, South Africa. Data were gathered via four participant groups viz. Deaf learners, their parents, teachers and officials of the Department of Basic Education, as well as through document reviews.

**Results:**

The results indicated that multiple linguistic exclusions for these learners begin early in their lives and continue into their school years and beyond. These experiences at home, school and in social contexts combined impact negatively career construction and its prospects.

**Conclusion:**

This study concluded that linguistic exclusions experienced by Deaf learners are created by a combination of systemic factors, which impede the career construction of D/deaf learners. Implications and suggestions for advancing their linguistic inclusion are discussed.

## Introduction

Since the Salamanca Statement in 1994, the international discourse in education has focused on inclusive education (Pather 2019; Salamanca 1994). Inclusive education was aimed at combatting discriminatory attitudes and the development of inclusive communities to achieve education for all (Pather 2019). This statement was made in the same year when South Africa as a country obtained its democracy. However, 27 years post-democracy in South Africa, D/deaf learners continue to be marginalised. Learners who are D/deaf or severely hard-of-hearing who use Sign Language as a communication medium, in particular, have been provided little attention in the aspect of inclusion because of their relatively small number and low visibility (Csizér & Kontra 2020).

The South African Department of Basic Education refers to education as a tool to address previous inequalities that were imposed by the apartheid government. The apartheid regime introduced segregation among racial groups, which included schools. Most relatively affluent white communities had better-resourced schools and services (Storbeck & Martin 2010), and remained in big cities with good infrastructure, while the black people were forced to live mostly in the homelands, which were not considered to be part of South Africa. This segregation negatively affected the resources of the schools for^1^ Deaf people in the former homelands where this study was conducted. In order to address this imbalance, the government’s redress strategies were designed to empower South Africans with equal education (The Constitution of the Republic of South Africa 1996). People of South Africa, including persons with disabilities, must be empowered through education to facilitate their socio-economic freedom. The Department of Basic Education in South Africa envisions that all South Africans should have access to lifelong learning, education and training opportunities. This would, in turn, contribute to improving the quality of life, thereby building a peaceful, prosperous and democratic South Africa (Department of Basic Education 2017). In the recent speech of the Minister of Basic Education, it was mentioned that the Ministry of Basic Education believes that an inclusive education system makes an immense contribution towards an inclusive economy to serve an inclusive society (Motshekga 2022). Providing learners with special education needs gives access to quality basic education programmes, and is found to be imperative based on the constitutional social justice principles of equity, inclusivity and redress. Education is a necessary tool to bring about change in a society, the economy and the ideologies of the world (WFD 2018). Education imparts abilities, information and recognition, which positively influence employment and earnings (Kelly, Quagliata & Perotti 2016). Deaf people must satisfy the same educational attainments as hearing people to compete in the job market (Kelly et al. 2016).

The literature shows that inevitably, compromised text literacy skills displayed by Deaf adults impact the education and employment of Deaf school leavers (Glaser & Van Pletzen 2012). This situation could be prevented by providing education for Deaf learners on par with that offered to hearing children through equal language access. Deaf learners can fully access education through a signed language, and their educational needs can be most efficiently, equitably and cost-effectively met in South African Sign Language (SASL) centres where schooling in all subjects would be provided through the medium of a signed language (Aarons & Akach 2002:153 in Glaser & Van Pletzen 2012). In education, language plays an important role in imparting knowledge. The Minister of Basic Education mentioned that the programme of early childhood development offers the foundations for lifelong learning where learners are exposed to in 2009, included language, motor skills, perceptual skills, problem-solving, basic numeracy, self-regulation, executive functioning and the love for learning (Motshekga 2022). However, this programme was referring to the majority of learners with no hearing impairment who attended matric examinations in 2021. In the speech, there was no mention of inclusive early childhood development programme, which would provide the desired educational outcomes for learners who are D/deaf nationally.

The purpose of this study was to explain the linguistic exclusions faced by learners who are deaf in the Eastern Cape of South Africa. The term ‘D/deaf’ is used in this article to acknowledge the cultural Deaf and clinically deaf persons concurrently. This is also to avoid making assumptions about the identity choices of persons with deafness. D/deaf learners in the Eastern Cape province require good education for economic inclusion and participation. By highlighting these exclusions, the authors hope that policymakers and implementors will develop strategies to reduce or eliminate exclusions. The lessons learnt in this case study may find resonance with other countries, which may share similarities with South Africa. The knowledge generated contributes to the research already available on secondary and post-secondary education and training for D/deaf students in African countries, which is described as very little (Glaser & Van Pletzen 2012).

According to the literature, exposing D/deaf children to Sign Language early in life has positive developmental and educational benefits (Hall, Hall & Caselli 2019). On language ideologies and epistemic exclusions, Kiramba from Kenya argues that teaching in one language only and not accommodating home languages for non-English speaking learners in schools exclude them in education. Considering other languages offers an inclusive alternative to education approaches (Kiramba 2018). In Zimbabwe, Musengi argues that if Deaf people are supported in using Sign Language for communication, then they should be permitted to receive education in Sign Language, otherwise they are being discriminated against through ‘audism’ (Musengi 2019). We already know from the literature that there is^2^ ‘a critical period’ (birth to early teenage years) for language development for children, in general; however, D/deaf children born to hearing families usually pass this period without being exposed to spoken or signed languages (Cheng, Halgren & Mayberry 2018). This lack of linguistic exposure affects the educational trajectories of these children and subsequently their career construction. The literature does not offer specific information on how D/deaf learners’ career construction is affected by linguistic exclusions. This study contributes to the knowledge gap in the effects of linguistic exclusions experienced by D/deaf learners in the Eastern Cape of South Africa’s career construction.

## The theoretical framework

The theory of career construction developed by Savickas in 2001 provided a lens that framed this article, which addresses and explores the career construction of deaf learners in the Eastern Cape. The theory of career construction explains the interpretive and interpersonal process through which individuals impose meaning and direction on their vocational behaviour. The career construction theory addresses how the career world is crafted through individual^3^ constructivism and social^4^ constructionism (Savickas 2005). It emphasises that individuals create representations of reality but do not make reality itself. Careers do not unfold; they are constructed as individuals make choices that express their self-concepts and substantiate their goals in the social reality of work roles.

This theory has stimulated the current study, as its philosophical position and assumptions align with the interests of the researcher and have guided in knowledge generation.

Career construction was found to be a powerful framework that promotes the self-determination of D/deaf learners excluded through language (Kwon 2017). The career construction theory accepts that people are different, and that acceptance includes people with disabilities in career construction. The theory can enhance adaptations that can improve how environments can allow persons with disabilities to access careers of their choice (Rudolph, Zacher & Hirschi 2019). This study focuses on the matrix of exclusions for D/deaf learners with a special interest in linguistic exclusions. The study explores the role played by D/deaf learner individuals and the context in the matrix of exclusions. The findings were interpreted against the assumptions of this theory. The theory mentions a dichotomy of conditions to be met for successful career construction: these are individual constructivism and social constructionism. It will be instrumental in exploring whether the dichotomy conditions are met and may assist in describing what contributes to these being met or not in the study context. The theory also assists in revealing how these conditions influence each other, how one would not succeed with individual constructivism if the environment hinders social constructionism.

## The context

The context in which Deaf learners from the Eastern Cape find themselves is defined by the adverse effects of historical colonisation, where global colonialism resulted in economic oppression and the general underdevelopment of the Global South. The goals of global colonisation were to exploit and dominate the people and resources of the Global South (Maldonado-Torres 2018). While the colonial administration ended in South Africa in 1961, the apartheid government ruled from 1961 to 1994, which deepened racial discrimination in the education system even further. While official colonisation and apartheid ended, South Africa continues to experience the impacts of colonisation (Oliver & Oliver 2017). Although the era of colonialism is long gone, its effects are still evident more so in the former homelands and their institutions. Mashau refers to this colonial phenomenon as a dead snake that continues to raise its head through public squares and churches. He says South Africa as a country still feels the hangover of the past apartheid (Mashau 2018).

This study was carried out in the Eastern Cape, a province created in the post-apartheid period, when South Africa was divided into nine provinces. The Eastern Cape province of South Africa is rated as the second poorest province in the country (Stats SA 2011). Most of it is a previous homeland, Transkei, suffering high rates of poverty, unemployment and disability. The province is predominantly rural, mostly composed of black South Africans. The children referred to in this case study predominantly come from the deep rural areas, semi-urban and townships. Children from this background receive a poor start to life compared with those in the more affluent parts of the country and the world. This disparity is associated with the political and socio-economic history and the under-resourced education system in this province. Their poor start affects their education and career trajectories, which later negatively impacts their economic activities and contributions. In comparison with other provinces in the country, the Eastern Cape performed poorly in education (Stats SA 2016). It is argued in this study that the linguistic exclusions in education experienced by D/deaf learners are influenced by their context.

The case study was conducted in 2020 in two of the high schools, which serve the Deaf population of the Eastern Cape. From the post-apartheid era to date, 47 schools serve the Deaf population in South Africa. In the Eastern Cape, there are four public schools for the D/deaf, with all these schools starting from the foundation phase and taking learners through to matric. Two of these four high schools were included in the study. The settings of these high schools are different, one in a more rural setting and the other in a semi-urban setting. The resourcing of these high schools was very similar. From the literature, we already know that in South Africa communication support for D/deaf education is pivotal and yet lacking (Ngobeni, Maimane & Rankhumise 2020), leading to a high dropout rate in education. D/deaf children need linguistic support from home, school and beyond, which would grant them linguistic inclusion and enable them to achieve their career aspirations better.

## Aim and objectives

This study aimed to highlight linguistic exclusions experienced by D/deaf learners in the Eastern Cape. The objective was to describe the multiple facets of linguistic exclusions experienced by D/deaf learners in the Eastern Cape and their possible causes.

## Research methods and design

### Study design

This study adopted a qualitative research methodology (Creswell 2007). A descriptive case study design was adopted because the researchers wanted an in-depth understanding of the factors influencing the career construction of D/deaf learners.

### Study population and sampling strategy

The study population included four participant groups: the D/deaf learners from high school who were enrolled in grades 10–12 (*n* = 38), their parents (*n* = 19), the teachers involved in the career guidance of the learners (18) and members of the Department of Basic Education responsible for career guidance in deaf high schools (*n* = 3). The documents for review were five (*n* = 5). A purposeful sampling strategy was used.

### Data collection

The data were collected using multiple methods, which included focus group discussions, individual interviews (face-to-face and telephonic) and document review. The multiple methods of data collection reduced and neutralised the bias and weakness of using the same data collection method (Creswell 2013).

## Data analysis

Thematic analysis was used to analyse the findings and generate themes to guide the discussion of these findings (Nowell et al. 2017). Themes were formulated considering the aims and objectives of this study. The data were organised according to the different settings where linguistic development is expected to reflect. The data were analysed using a three-level synthesis to understand the phenomena of multiple linguistic exclusions in this case study (Vaismoradi et al. 2016). In the end, themes are abstracted using the theory of career construction and its concepts of individual constructivism and social constructionism to make meaningful knowledge production.

### Data representation

The data are presented as direct quotations from the participants. The quotes of participants included in this article were selected to represent all participants and all areas of discussion and interviews related to the linguistic challenges that influenced how careers were constructed.

### Ethical considerations

The study was conducted in accordance with the Helsinki Declaration (Ethics approval No. HERC REF:015/2018). The operational guidelines as per the ministerial consent for non-therapeutic health research with minors of 2015 were also considered. Learners who were under the age for consenting signed an assent form instead. Participants were given consent letters that explained all the details including the freedom to withdraw at any stage of the study with no penalty involved.

## Findings

In this article, linguistic exclusion is presented as a theme representing multiple ways of exclusion. The linguistic exclusion was found to be a critical form of exclusion that is a challenge for D/deaf learners in this context. We present language exclusion within different settings as discussed below.

### Linguistic exclusion

This case study has identified exclusions of D/deaf children in language, socio-economic, curriculum, career information, extra-curricular, sports and policy implementations. However, the most challenging of these exclusions and the one that has a significant impact on education and its outcomes is linguistic exclusion. This exclusion occurs in multiple settings for the learners, at all life stages beginning from early life to adulthood, and this, directly and indirectly, excludes them from education.

They are excluded in learning Sign Language before entering the school, during school years and throughout life.

### Home settings

Parents of the learners in this study have communicated their inability to communicate with their children.

One of the parents said:

‘[*T*]he problem is that my child speaks in a difficult way and I cannot speak to her. I don’t know if she speaks to her friends and sisters because it is them who are able to speak with her in detail.’ (Parent no. 5, female, interview group 1)

In most instances, parents do not know from birth that their children are deaf. The children usually get diagnosed late, mostly at an age of 4 years, where they should be going to pre-school already. Children diagnosed with hearing loss are placed in suitable schools late generally due to lack of early identification and intervention programmes in the Eastern Cape. Parents struggle to accept that their children are deaf due to the social stigma associated with deafness or disability, in general. All the dilemmas reported in the home settings by this contribute to the language deprivation and exclusion of these learners.

A teacher expressed this concern:

‘Some parents delay acceptance of the deafness of their children, they become overprotective and do not teach or communicate to their children. They fear things that may happen to their children when they are not in their sight.’ (Teacher no. 2, female, focus group 2)

IsiXhosa is the home language used mainly in the Eastern Cape and in the homes of the participants; however, isiXhosa is inaccessible to these children because they are deaf, and Sign Language is inaccessible to parents because they have no way of learning it, especially before they know they have a child who may not communicate in their home language. It was also noted during the study that parents from this region have no available support to assist them in learning Sign Language for communicating with their children, especially before they are placed in schools. This linguistic support limits language learning for D/deaf children.

Although parents expressed their interest in learning Sign Language, they were unable to do so because they live far away from the schools, which could be resource centres for them to learn Sign Language. Moreover, their children attended and stayed in boarding facilities of schools for D/deaf children, so the parents would not have much opportunity to use Sign Language with them. The learners in the study communicated their agitation by the following expression:

‘No that does not happen. They do not know Sign Language. They only talk; our parents do not know Sign Language. No, we do not talk to them. Our parents do not know Sign Language; they must be called into the school hall and be taught Sign Language so we can communicate with them.’ (Learner no. 7, male, focus group 1)

### Pre-school settings

The system of education does not have public pre-school facilities to prepare D/deaf children for formal education in the Eastern Cape. One of the teachers reflected a sense of disquiet saying:

‘There are no pre-schools for deaf children and therefore no pre-school preparation. They start late in schools aged ± 12 years and they must do grade one or grade R. At that age, they have to start acquiring language, which is Sign Language. They only get their names in schools and we know language is important for education. When finally in school now they struggle with education concepts.’ (Teacher no. 3, female, focus group 2)

The teachers and education officials in special needs education have explained that one of the challenges in educating D/deaf learners in the Eastern Cape is the children entering school late in terms of their age, with no established language for communication. This pre-school exclusion together with limited home language support affects the education of D/deaf learners. Another teacher said:

‘Deaf children acquire language very late and this leads to isolation. They are called by names like “isimumu” [*which means the mute one*] and they are shamed. This isolation and shaming contribute to the parents and children’s lack of confidence to socialise early and acquire language early.’ (Teacher no. 1, female, focus group 2)

This concern was emphasised by more teachers who said:

‘The school needs some orientation classes to the language of the school because this creates a gap in teaching and learning.’ (Teacher no. 3, male, focus group 1)

‘The problem is poor foundation and this is frustrating to teachers.’ (Teacher no. 5, male, focus group 1)

‘Ideally a child needs to get into a deaf school at age 4 but we are finding that we are getting them there very late in their lives, they have already missed out. We also know that critical age of language acquisition is 3 to 7 years. If you admit a child and introduce a new language to them at age 8 to 9 then it becomes a problem you are setting that child up for failure for a lack of a better word because now in addition to having to give input in terms of language, teaching them language rules you also need to teach them in that language that they have not mastered from a very young age, so automatically the child because of systematic problem has been deemed that they will have academically a problem.’ (Official no. 3, female, interview 3)

The findings in this pre-school phase setting attest to the structural exclusions because of the unavailability of pre-schools, which could be a resource for early linguistic exposure to D/deaf learners and their parents. The early childhood deprivation of these learners tends to disadvantage them when compared with their hearing counterparts who have access to pre-schools and proper preparation for schooling and education. D/deaf learners enter schools with poor linguistic skills, which are crucial for learning the educational content. A number of research studies have proven that early language development is important for education. If the child has inadequate language, he or she practically has no means of communication, and this includes gaining an education.

### During school settings

Linguistic exclusion is also experienced during schooling years, while they are in D/deaf schools, which should cater for their linguistic needs. In the Eastern Cape, D/deaf schools are still segregated from mainstream schools. In these schools, D/deaf learners are mostly taught by teachers who do not know Sign Language, some of whom did not receive basic Sign Language training. A teacher said during the focus group discussions in one of the schools:

‘I just got employed to teach life skills and I was happy to find work, but at the college, I was never given a chance to practice on deaf learners. There is not even a module that covers teaching in special schools or special populations. The Sign Language I know I have been taught by learners. I was employed in 2016 but got my first Sign Language training 2 years later in 2018. There is no induction even from the school.’ (Teacher no. 5, male, focus group 3)

Another teacher said:

‘The Sign Language we have is either self-taught, taught by learners or taught by asking assistance from other teachers.’ (Teacher no. 7, female, focus group 2)

A third teacher said:

‘There is a barrier while teaching I have to convert English to Sign Language so I can be able to transfer the knowledge.’ (Teacher no. 4, female, focus group 1)

This lack of knowledge of Sign Language by educators leads to inadequate delivery of educational content. The next teacher said:

‘There is a lack of deaf teachers; the teacher aides have limited knowledge of subjects’ content.’ (Teacher no. 1, male, focus group 1)

Most of the teachers in these schools are isiXhosa home language users. English, which is the language used to deliver curriculum content is their second language, then Sign Language becomes the third or fourth additional language to them. During training, English is the assumed language for learning and teaching, and these teachers have to teach the curriculum content in a language that is their second language. It becomes more challenging for them to have to translate the educational content to the third language, which they are learning while they are teaching. This complicates matters for both teachers and learners, and this underpins the linguistic exclusion during school settings.

The teachers are also not trained on how to educate learners with disabilities despite the availability of policies that prepare the platforms for inclusive education like White Paper 6 and SIAS (Department of Basic Education 2001). The evidence to support this is when a teacher said:

‘The teachers are not capacitated to deal with learners who have disabilities especially with multiple disabilities.’ (Teacher no. 3, male, focus group 3)

They also mentioned that in their training they were never capacitated to adapt the general curriculum to Sign Language, and this causes barriers in the completion of the curriculum designed for each grade:

‘The curriculum has not been adapted to suit the learners who are deaf. The teachers are doing the adaptation and this delays the finishing of the curriculum, it does not get finished.’ (Teacher no. 2, female, focus group 3)

The teachers also expressed concerns in the assessments of D/deaf learners. Due to the different structure of Sign Language to the English language structure (which is a language of curriculum delivery) learners are assessed unfairly. The teacher said:

‘Another challenge is the language. The language structure of Sign Language and spoken language is not the same. The deaf people write as they talk and this becomes a barrier when they are being assessed.’ (Teacher no. 2, female, focus group 3)

Another teacher shared the same concern and said:

‘The Department of Education conducts workshops on Sign Language but the ability of the teachers to use Sign Language also contributes to curriculum delay, we are not able to finish in time. In the exams, there is no Sign Language interpreter for deaf learners and poses a challenge during exams.’ (Teacher no. 3, male, focus group 3)

The learners also expressed their difficulties with the language in their education. One of the learners said:

‘The way we are taught is bad, our teachers speak with their mouths, they write on the board, they move their lips and we are not able to hear. There is no Sign Language and we are deaf at that time.’ (Learner no. 13, male, focus group 1)

This expresses the reality of how they are taught, where teachers would be speaking and writing in languages that are not understood by learners. Teachers may have been doing this because of their frustrations on the language of communication; however, it brings more frustration to the learners. Another learner said:

‘Here at school, we are taught subjects like for example English, and we don’t know English, maybe technology and we don’t know technology but its taught, we don’t think we will go to university, we don’t know English, we don’t know even that technology we don’t think we will get to university.’ (Learner no. 3, female, focus group 1)

This was referring to the exclusion in academic content being delivered in a language that learners cannot access by both hearing and reading. Another one said:

‘English words are difficult, we do not understand it, and it is very difficult.’ (Learner no. 9, male, focus group 1)

The difficulty expressed here could mean that they cannot speak it or write it. Maybe, the learners experienced the English language’s linguistic rules to be difficult to learn because the grammatic rules are different from Sign Language, and therefore, it is experienced as being difficult. For example, another learner’s input was as follows:

‘For example, teachers write and write on the board, we understand nothing in it, no interpretation of what is written, it is just writing.’ (Learner no. 4, male, focus group 1)

‘No the education we are getting here is very ugly and it is boring.’ (Learner no. 5, female, focus group 1)

‘We do not get interpretations, we bear the pain, the teachers just come and write and we copy what is written.’ (Learner no. 1, male, focus group 1)

This means that there is no learning happening in the classrooms, as the learners just copy what they do not understand and what has not been interpreted in a language and manner that they follow and understand. The adjectives used by learners in the statements above (ugly, boring, pain(full) or bear the pain) show that there is frustration on the side of learners too concerning the language used in teaching. This is a clear indicator of linguistic exclusion in a school setting. Learners further suggested alternative ways, which they believed are more inclusive for them to learn, like technical skills training and teachers getting training in Sign Language. Learners said:

‘We want education with technical training, it will make us understand, and we are just bearing with the one we are getting.’ (Learner no. 4, male, focus group 1)

‘The teachers must regularly go for Sign Language workshops, they must be taught Sign Language.’ (Learner no. 14, female, focus group 1)

An example of how themes were developed during data analysis to make sense of the findings is displayed in Table 1.

**TABLE 1:** An example of themes developed during data analysis.

Aim and objective	Settings	Direct quotes (raw data)	Coding (synthesis 1)	Meaning-making (synthesis 2)	Themes development (synthesis 3)
Aim: To highlight the linguistic exclusions experienced by D/deaf learners in the Eastern Cape.	Home	**Parent:** ‘[*T*]he problem is that my child speaks in a difficult way and I cannot speak to her. I don’t know if she speaks to her friends and sisters because it is them who are able to speak with her in detail’.	Cannot speak to her	Barrier in communication is caused by a delayed language of communication development	Language of communication
Objective: To describe the multiple facets of linguistic exclusions.		**Teacher:** ‘Some parents delay acceptance of the deafness of their children, they become overprotective and do not teach or communicate to their children. They fear things that may happen to their children when they are not in their sight’.	Do not communicate	-	-
		**Learner:** ‘No that does not happen. They do not know Sign Language. They only talk; our parents do not know Sign Language’. ‘No, we do not talk to them’. ‘Our parents do not know Sign Language; they must be called into the school hall and be taught Sign Language so we can communicate with them’.	Do not know Sign Language	-	-
			Must be taught Sign Language		

The figure represents the four themes from the findings that expose the developmental links to the matrix of linguistic exclusion for the D/deaf learners in the Eastern Cape. It also shows how the themes are linked with the theory of career construction to explain it.

## Discussion

**FIGURE 1:**
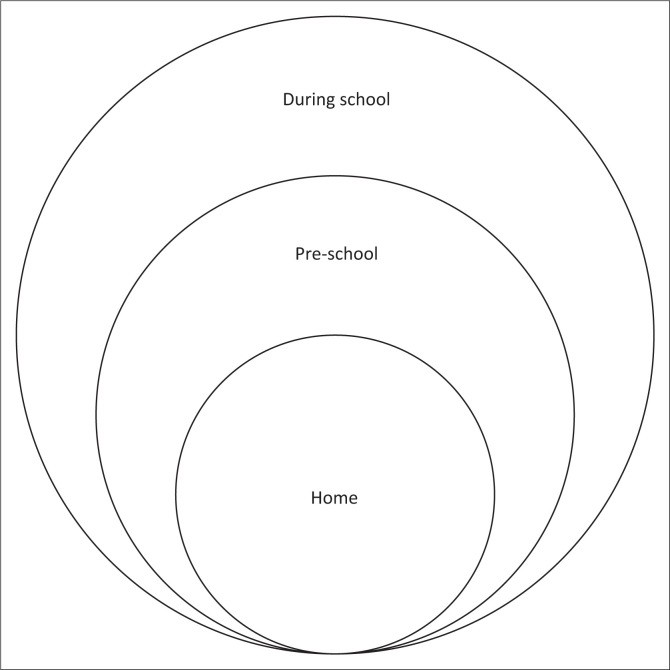
Graphical representation of contexts of linguistic exclusion experienced by D/deaf learners.

**FIGURE 2:**
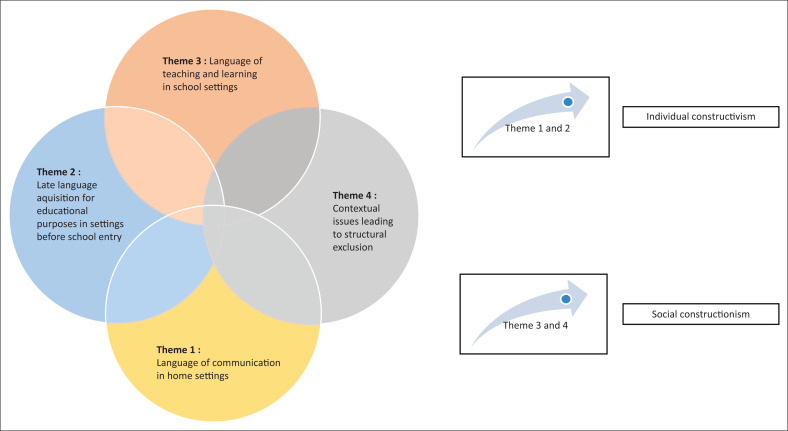
The developmental links to the linguistic exclusion matrix.

This case study has revealed multiple linguistic exclusions for learners who are D/deaf and residing in the Eastern Cape, as well as contextual issues that worsen these exclusions. It has also shown the cumulative effect of the layers of linguistic exclusion from early life through school years and later into adult life. Figure 1 is a representation of linguistic contexts where linguistic exclusions occur for the learners in this study. The context where these learners come from was also instrumental in the lack of support that they experienced. The exploration of the two constructs of career construction theory has shaped the understanding of this study. The first construct of individual constructivism was affected by several issues that are discussed below. The language was important and seemed to be a barrier to individual constructivism. The second construct of social constructionism was also explored and seen as lacking due to contextual barriers. This discussion is organised according to the themes that comprise the matrix of exclusions and the career construction theory used as a framework against which the themes were developed. Figure 2 represents the themes that link up to develop the layers of linguistic exclusions for the learners.

### Language of communication at home settings

The delayed language development from home had an impact on later stages of life in these learners. Communication is important for making contact, reaching out to others, satisfying our needs, revealing feelings, sharing information and accomplishing many things (Owens, Farinella & Metz 2015). When there is a barrier in communication, it is impossible to fulfil communication needs. Language is a socially shared code used to represent concepts (Owens et al. 2015). When we do not share the same language, it is challenging to communicate effectively. A barrier in language translates into less effective communication. Communication between learners and parents in home settings was inadequate in the early lives of these deaf children because of the language gaps imposed by deafness and the unawareness of the parents about alternative ways of communicating with their children who cannot communicate through spoken language. These learners did not have access to Sign Language early in their development and could not access oral languages like isiXhosa, which is the language spoken at their homes. The missed opportunity to develop or learn a language at home before school is an exclusionary linguistic factor that generally impacts their socialisation as well as their education career trajectories.

The home settings become the inner layer in linguistic exclusion as the children navigate through childhood development to school. When the children entered schools, they brought an inadequate linguistic foundation to a context that assumed linguistic preparedness. In this case, this preparedness did not exist. The home language for D/deaf learners in this study is isiXhosa, the parents and hearing siblings speak this language. Deaf children cannot access the spoken language because of deafness, and therefore, they do not develop their home language and cannot access it either. This lack of linguistic exposure in early life leads to delayed language development as children were not supported to develop language skills at critical ages.

The development of language is key in developmental milestones. It involves a series of steps, such as bodily growth, for being able to express it, cognitive preparedness to receive and interpret it. The availability of good language models to imitate and practice with and also for encouragement and guidance is crucial (Ritonga & Sofyani 2018). For these learners, some important language supports were not available for language acquisition. Language development in early childhood is important for early learning and developing social skills (Owens 2014). Children learn the language first and later use the language to learn in schools; this is a normal development in terms of milestones (Owens 2014). For these learners, the poor linguistic foundation is a deprivation that contributes to the early exclusions, which later negatively impact their education. The linguistic support that could help these children would be the availability of support for their parents to have an alternative language to communicate. The benefits of early access to Sign Language or any natural language lead to believing that the most effective way to reduce language deprivation in children generally is to expose them to, and immerse them in, language as early as possible in their development (Hall et al. 2019). The suggested early linguistic exposure lays a good foundation for preparing children for the next milestone of life, which is to be prepared for schooling.

### Late language acquisition for education in settings before the school entry

The findings revealed that teachers and educational officials were greatly concerned by the level of linguistic unpreparedness that D/deaf learners presented with when they entered the school. They were also concerned about the ages at which children are enrolled into schools for the first time. The effects of linguistic unpreparedness had negatively impacted on the education of the learners and the teachers. The linguistic unpreparedness for schooling becomes an early exclusion for D/deaf learners before entering schools. This is the second layer of exclusion from language leaning experienced by D/deaf learners in the Eastern Cape in the early years. Their late language acquisition delays their education progress because when children enter school, they only learn their language basics at a school-going age (±7 years). Instead of learning the grade 1 content, learners must still learn their Sign Language names and basic communication like greetings, turn-taking, etc. In hearing children, who have been to pre-schools the picture is very different. Late language acquisition becomes an excluding factor in effective foundational education. The next theme to be discussed is the influencer of late language acquisition and what happens in settings before the school entry.

### Contextual issues leading to structural exclusion

The context of the province was explained in detail in the early pages of this article to paint a clear picture of the context referred to throughout this article. The contextual issues are presented as the third layer of linguistic exclusions as it influences the linguistic outcomes. In the Eastern Cape, the first issue is that there are no preparatory schools for D/deaf children, when they enter the school, they are in an environment of learning with no preparedness for it, especially in language. When D/deaf children enter schools, mostly they come from home settings, they have skipped an important preparatory phase for education. When they enter schools for the first time, the teachers mentioned that it is when they receive the Deaf identity and are given their Deaf names. This milestone in development should have been achieved before the school; however, as there are no pre-schools for the D/deaf children in the Eastern Cape this does not happen. This challenges the process and progress of learning in classrooms.

The second issue is the late identification of hearing loss as mentioned by teachers. They mentioned that hearing loss is identified late by parents because of a lack of early identification and intervention programmes by the Eastern Cape government institutions. When the diagnosis of hearing loss is finally made, children are placed late in appropriate schools because of there being fewer specialists who should place the learners in suitable schools. This reflects on the poor resourcing of the government facilities. The late identification and placement of learners contribute to early exclusion for these learners because some are not able to catch up with school-age requirements. They reach adulthood while still in the lower grades in school, and this negatively affects their career trajectories and the construction of careers. The Health Professions Council of South Africa has a policy on Early Detection of Hearing Impairment in new-born babies. This policy is not implemented in this context. Reasons for this are not part of the focus of this study.

The stigmatisation of disabilities was another issue revealed by teachers, which affect parents in seeking early support for diagnosis and early school placements. This stigmatisation is associated with a lack of awareness and available information on disabilities, leading to delayed support-seeking behaviour by parents. Children who are D/deaf and their parents miss out on the early support necessary for their communication and livelihoods. It is that early support that learners from more advantaged contexts may have, which assists in preparing and including them in education. The learners suggested that their parents should be taught basic Sign Language so that they can communicate with them. The parents expressed their interest in receiving such training; however, in this context, there is a lack of facilities to train the parents in Sign Language. This deprives both the parents and children of a chance to learn to communicate effectively, thus leading to educational complications and career construction difficulties.

### Language of teaching and learning in school settings

The mode of delivery of education content in the curriculum to learners in South Africa is mainly English. The learners in this study have expressed difficulty in learning or accessing the language of instruction in education. This is mainly caused by their inability to access verbal forms of communication because of poor hearing, coupled with their challenges in becoming literate in English. This is the fourth layer of linguistic exclusion in this case. The teachers of D/deaf learners are not first-language speakers of English, and they have a challenge of modifying this second or additional language curriculum content into another language, being Sign Language, that is accessible to the students. The findings reveal the challenges experienced in the delivery of language of curriculum. The challenge cuts even deeper in this case where both teachers and learners do not share a common language of communication. The teachers use isiXhosa as their first language, while the learners use Sign Language as their first language. English is an additional language for both but is a legitimate language of instruction in South African education. Failure to understand and use it means a general failure in education (Kiramba 2018). This language mismatch leads to a failure in delivery of and receiving the educational content. The consequences that result from this is poor construction of careers stemming from displeasing educational outcomes. Non-legitimisation of Sign Language as a language of instruction for D/deaf learners is excluding this category of learners from education.

The South African Department of Basic Education, in its endeavours to remove the linguistic marginalisation and to improve deaf education in South Africa, has developed and published a policy named ‘The Curriculum and Assessment Policy Statements (CAPS) for South African Sign Language (SASL) Grades R-12’. This policy aims to remove the barrier of language in their education as it proposes that D/deaf learners must be educated in Sign Language, which is applauded as a step in the right direction by the Deaf education activists (Department of Education 2014). The policy introduces a new moment in time in the education encounters of D/deaf learners in South Africa (Department of Basic Education 2014). However, this policy implementation had not commenced in 2018 at the schools studied, which was four years after its development. The availability of the policy does not suffice, and it is its implementation that will make the difference in the inclusion of D/deaf learners.

The South African Department of Education also made provisions following its Education white paper 6 of 2001. The focus of this study was on inclusive education, the policy on screening, identification, assessment and support (SIAS 2014). This is a strategy that facilitates early identification and placement of students to include them in education. However, these strategies have not benefitted these learners as their implementation has not, and is still not, happening as it should.

In recent developments, the South African parliament has approved the amending of its Constitution to make South African Sign Language the 12th official language (Maqhina 2021). This progress is widely welcomed by the Deaf population, with the hope of improving the current status of linguistic exclusion in education and other social contexts in South Africa. The policy frameworks for inclusive education are slowly being established in South Africa, although in a context like that of the Eastern Cape inclusive education is still not fully met.

The findings of a South African study conducted in Motheo district, at a school for the Deaf and Blind, concurs with the findings of this study. It highlights the poor training of teachers in SASL, late language acquisition by Deaf learners as well as a lack of physical resourcing of the schools. All of this contributed to barriers for learners in grade 8 (Ngobeni 2017).

A study on inclusive education for Deaf learners in Zimbabwe also concurs that full inclusion of Deaf learners can only be experienced when they have access to their preferred language which can be visualised (Musengi 2019). International studies in this area affirmed that language is a critical factor contributing to exclusion of deaf learners in education. Inclusive education is echoed many times as a means of ensuring the education rights of D/deaf people, as outlined by the United Nations Convention on the Rights of Persons with Disabilities (Jokinen 2018). The concept of inclusive education may allow multiple meanings and may not be suitable for deaf children because they are still linguistically and socially excluded, and this does not assist their education (Kusters et al. 2015). Inclusive education for deaf people has been further defined as ensuring that education is provided in their first language using suitable modes and methods of communication for the individuals and spaces. These should maximise learning and general growth (Jokinen 2018). For deaf children, full inclusion is equivalent to a fully supportive, manual communication and learner-oriented environment. This provides a rich environment for academic growth and the necessary development socially and emotionally. For D/deaf children to experience their full potential in life and education, they must have access to Sign Language as this enhances, rather than reduces, their abilities (Moores 2018). However, this study reveals that such critical conditions of inclusion are not met. Internationally, scholars in this field concur that fully accessible language experiences during early childhood are important to develop the potential of D/deaf children (Hall et al. 2019).

The theory of career construction brings the two conditions of individual constructivism and social constructionism into play. The findings reveal a lack in both components of career construction but seem to reveal more weakness in conditions for social constructionism, which has impacted negatively on individual constructivism. When we discussed the themes from the matrix of linguistic exclusions, it seems that contextual issues have a huge negative effect on the education and career construction of D/deaf learners in the Eastern Cape. The linguistic matters reflect contextual and individual issues, and the systemic issues are reflecting the contextual influences. The unavailability of facilities for Sign Language training for parents of Deaf children, the lack of pre-schools for Deaf children, the lack of training for teachers of Deaf children, and the late identification and school placements for these children are all contributors to the social barriers to career construction. These are a reflection of risk factors to social constructionism. Even if these learners would aspire to formal career trajectories, they will always be held back by social inadequacies from achieving these, until these gaps are attended to. The matrix of exclusions will always exist until social and political influencers are addressed. The linguistic issues and general educational development of learners are reflecting a risk to individual constructivism because the language to join learning experiences in and out of school settings is key to individual constructivism, and this linguistic gap contributes to the matrix of exclusions. The multiplicity of exclusions arises from these matters. If this study was carried out in a different context in the world, the results may be different.

## Strengths and limitations

The concept of linguistic exclusion for D/deaf learners may not be new in the literature; however, this study reveals that D/deaf learners from the Eastern Cape in South Africa are trapped in a matrix of linguistic exclusions, which are mostly influenced by the context. The context was influenced by historical socio-political influences, resulting in deep inequalities It is this matrix of exclusions that contributes to undesired career construction outcomes.

The multiple methods of data collection and variety of participants offer strong rigour and validity to this study.

The limitation of the study is the case study design, which limits the generalisation of the findings to a context beyond the Eastern Cape of South Africa. The reader should consider not generalising these results to the larger population but rather to consider how the findings could be transferable to other contexts.

## Recommendations

The education system currently, although it is internationally advocated to be inclusive, is greatly challenged by what occurs before D/deaf children come into the system’s custody. In order to curb this influence, the authors suggest that early life interventions be considered to improve the inclusion of all learners in education. Interventions could include enforcing the implementation of an Early Hearing Detection and Intervention (EHDI) policy, which would lead to early identification and intervention in D/deaf children and families. This also includes advocating for early childhood development centres, which will provide D/deaf children and their families with the necessary early support, especially for language development and preparation for school. These interventions should lead to better educational outcomes, career construction and livelihoods for D/deaf communities. Implementation of the SASL CAPS curriculum is also critical in this case. Teacher development, especially for teachers who are placed in Deaf schools, is also critical, they must be skilled to teach in Sign Languages. Interventions need to consider the multiplicity of exclusions and the stages of life that they present at. A strategy has to be systemic and calls for a joint effort of the different sectors and government departments who are custodians of children at all stages of life through to adulthood.

## Conclusion

This study has discussed the cumulative effects of the matrix of linguistic exclusions. The linguistic exclusion for D/deaf children starts from early stages in life through to adulthood in multiple settings. As a result, in the Eastern Cape, the career construction is impeded by the combination of individual and social factors.

## Footnotes

1 Capital letter ‘D’ denotes a person with physical deafness and taking deafness as a cultural identity. A small letter ‘d’ denotes a person with physical deafness but not necessarily taking up deafness as their culture.

2 Critical period of language development (Robert 1997; Salwa 2019; UKEssays 2018).

3 Constructivism is ‘an approach to learning that holds that people actively construct or make their own knowledge and that reality is determined by the experiences of the learner’ (McLeod 2019).

4 Constructionism is more of an educational method which is based on the constructivist learning theory. Levels here are shifting from the physical (constructionism) to the mental (constructivism), from theory to philosophy to method, from science to approach to practice (Šverko & Babarović 2019).
